# ChimeraMiner: An Improved Chimeric Read Detection Pipeline and Its Application in Single Cell Sequencing

**DOI:** 10.3390/ijms20081953

**Published:** 2019-04-21

**Authors:** Na Lu, Junji Li, Changwei Bi, Jing Guo, Yuhan Tao, Kaihao Luan, Jing Tu, Zuhong Lu

**Affiliations:** State Key Lab of Bioelectronics, School of Biological Science and Medical Engineering, Southeast University, Nanjing 210096, China; nlu@seu.edu.cn (N.L.); ljjboris@gmail.com (J.L.); bichwei@163.com (C.B.); lebrowngj@126.com (J.G.); 220174561@seu.edu.cn (Y.T.); 213161290@seu.edu.cn (K.L.)

**Keywords:** MDA, chimeric sequence read, single cell sequencing, structural variation detection

## Abstract

As the most widely-used single cell whole genome amplification (WGA) approach, multiple displacement amplification (MDA) has a superior performance, due to the high-fidelity and processivity of phi29 DNA polymerase. However, chimeric reads, generated in MDA, cause severe disruption in many single-cell studies. Herein, we constructed ChimeraMiner, an improved chimeric read detection pipeline for analyzing the sequencing data of MDA and classified the chimeric sequences. Two datasets (MDA1 and MDA2) were used for evaluating and comparing the efficiency of ChimeraMiner and previous pipeline. Under the same hardware condition, ChimeraMiner spent only 43.4% (43.8% for MDA1 and 43.0% for MDA2) processing time. Respectively, 24.4 million (6.31%) read pairs out of 773 million reads, and 17.5 million (6.62%) read pairs out of 528 million reads were accurately classified as chimeras by ChimeraMiner. In addition to finding 83.60% (17,639,371) chimeras, which were detected by previous pipelines, ChimeraMiner screened 6,736,168 novel chimeras, most of which were missed by the previous pipeline. Applying in single-cell datasets, all three types of chimera were discovered in each dataset, which introduced plenty of false positives in structural variation (SV) detection. The identification and filtration of chimeras by ChimeraMiner removed most of the false positive SVs (83.8%). ChimeraMiner revealed improved efficiency in discovering chimeric reads, and is promising to be widely used in single-cell sequencing.

## 1. Introduction

**Figure 1:**
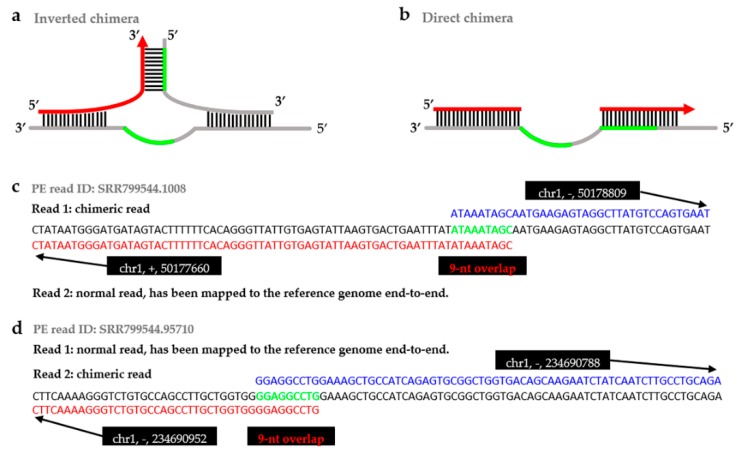
The mechanism of chimera formation and the example visualization of the single-end chimeras. There was branch migration reaction during the MDA processing, because of there exists same sequence (green line in **a**,**b**) on adjacent templates (grey line in **a**,**b**). (**a**) The displaced 3′-end annealed to other template where the sequence on the 3′-end (red arrow in **a**) had annealed to the sequence on the 5′-strand (green line in **a**), then continued elongation and formed inverted chimera; (**b**) The displaced 3′-end annealed to other template where the sequence on the 3′-end (red line in **b**) had annealed to the sequence on the 3′-strand (green line in **b**), then continued elongation and formed direct chimera. In (**a**,**b**), grey lines are DNA templates, black lines represent base pairing, arrows show the direction of elongation, and red lines represent chimeras; (**c**) The chimeric read parts mapped to the reverse strands (+/−), defined as inverted chimera; (**d**) The chimera’s parts mapped to the same strands (−/−), defined as direct chimeras. The green sequences in (**c**,**d**) are the overlaps of the chimeras, between the parts of a chimera. The red sequences are part 1 of a chimera, and the blue sequences are part 2 of a chimera.

Among the single cell whole genome amplification (WGA) approaches, multiple displacement amplification (MDA) [1] is considered a conventional approach to comprehensive applications, due to the efficient processivity, proofreading ability [2], and strand displacement activity [2,3] of phi29 DNA polymerase [2,4]. However, chimeric reads (i.e., chimeras) were discovered in the MDA sequencing data and caught more and more attention [5,6,7,8]. Chimeras resulted from alterative secondary structures, that can occur in the highly branched DNA, formed during the MDA processing [6], appearing as DNA rearrangements in amplified DNA (Figure 1a,b). The structure of chimeras is made up of two or more parts, which are located consecutively but adjacently on a chromosome (Figure 1c,d) [6,8,9]. In most bioinformatics analyses, chimeras can be filtered after mapping [5,10,11], but they still result in breaks in some researches, such as the ones aiming at structural variation (SV) detection [12,13,14,15,16,17], de novo genome assembly [18,19,20,21], and genetic recombination studies [22]. When identifying SV in MDA sequencing data, the formation of chimera will introduce false positive SVs [23,24], with an emphasis on inversions [25], will also increase the subsequent SV validation effort by >200-fold [25]. In addition, when constructing contigs for de novo genome assembly, chimeras will complicate genome assembly by linking non-contiguous chromosomal regions [21]. Therefore, elaborate analysis of chimeras is important for reducing these breaks.

The phi29DNApol-mediated chimeras were first discovered in 2006 [5], and were initially characterized by Lasken et al. in 2007 [6]. More comprehensive characterization was operated in 2015 [8] by exploring chimeras in over 130 giga-base MDA sequencing data [26]. A bioinformatics pipeline based on Short Oligonucleotide Analysis Package 2 (SOAP2) alignment software [27,28] was constructed at that time for Illumina HiSeq sequencing platform, to discover chimeric reads and to realize the statistics about the total amount of chimeras, as well as the detailed distributions of each kind of chimeras [8]. 

In previous works [8,9], authors divided chimeras into two categories: The insertion chimeras and the single-end chimeras. The insertion chimeras of the paired-end reads can be directly observed from the single-end mapped reads, based on the logical relation of mapping locations (+ or − strand). The bioinformatics pipeline for analyzing the single-end chimeras was established based on the subsection alignment strategy. Firstly, the paired-end reads were aligned to the reference genome, using SOAP2 [27,28]. The unmapped reads whose first 30-nt bases could map the reference were collected as the candidate single-end chimeras. Seed extension and local alignment were executed and circulated on the candidate single-end chimeras until the extended reads reached the upper limit of the mismatch. The reads were split into two parts from the tail end of the former part, and the lagging subsection were aligned to the 5000 bp local-regional of the former one, to find their exact location and the overlap sequences of two parts. Lastly, the single-end chimeras were divided into two types, direct chimeras and inverted chimeras, based on the two parts of a chimeras located at the reverse strands or the same strands. 

The bioinformatics pipeline above is complicated, the alignment and the circulation of extension take lots of time. In addition, the split-reads generated by the SOAP2 were not used for chimera analysis in the previous pipeline, which might miss plenty of single-end chimeras. Hence, we designed an improved bioinformatics pipeline to recognize chimeras from alignment results, and realized accurate classification and quantity statistics of chimeras, called ChimeraMiner. ChimeraMiner was built on the Burrows-Wheeler Aligner (BWA) [29,30,31], and the chimeras within single-end were recognized by using the soft-clipped alignment reads. Compared to the previous pipeline, ChimeraMiner was more efficient in chimera recognition and classification. Many chimeras, missed in the previous pipeline, were discovered using ChimeraMiner. Furthermore, we applied ChimeraMiner in several single-cell sequencing data for chimera discovering and evaluated the impact of chimera in SV detection.

## 2. Results

### 2.1. Data Downloaded and Initial Processing

We filtered the reads with “N” bases from all datasets, and the cleaned reads were used for alignment. Statistics of data and alignment results are provided in Table S1. Each sample had a high mapping ratio, revealing a well amplification during MDA.

### 2.2. Comparison between the Previous Pipeline and ChimeraMiner

We used both of the methods on two MDA samples to discover and classify the chimeras, SRX247249 and SRX252522 [26] (referred as MDA1, and MDA2, respectively). The duration of running was compared and the accuracy of ChimeraMiner was evaluated. When determining the valid single-end chimeras, we limited the subsection length no less than 30 bp. Moreover, we set the physical genome distance of two subsections ranging from 25 to 5000 bp, and the overlap sequence length longer than two bases.

As shown in Table 1, on the same computing platform, ChimeraMiner spent 60 h on MDA1 and 49 hours on MDA2 for alignment and chimera discovery, while the previous pipeline took 137 h on MDA1 and 114 h MDA2. The result demonstrated that ChimeraMiner was more efficient than the previous one. Using ChimeraMiner, 24,375,539 (about 6.31%) and 17,480,982 (about 6.62%) paired-end read pairs were ascribed as chimeras and classified into three types, which were both higher than the previous one. Many novel single-end chimeras were discovered. Most of them were attributed to the overlook of split-reads in the previous pipeline, and are investigated in the Discussion section. The results of the two pipelines demonstrated that the inverted chimeras are the dominant type of the single-end chimeras (average 91.36% by using ChimeraMiner, and average 88.69% by using the previous pipeline). The results were generally in concordance with the results of previous works [6,8,9], which endorsed that ChimeraMiner was accurate in chimera discovering.

**Figure 2:**
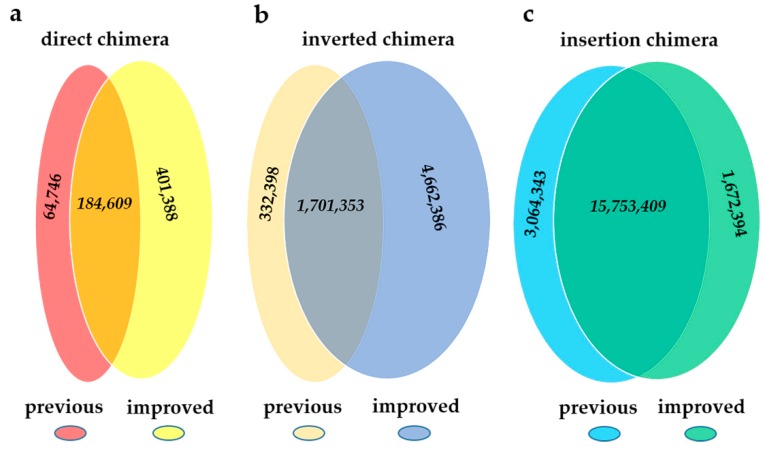
The comparison of two pipelines on MDA1. (**a**) direct chimera; (**b**) inverted chimera; (**c**) insertion chimera.

We further evaluated the coincidence of the results for two pipelines in sample MDA1, and the results were shown in Figure 2. For single-end chimeras, regardless of direct chimeras or inverted chimeras, ChimeraMiner found numerous novel ones, by contrast only a small number of chimeras was found by using the previous pipeline. The results of insertion chimeras were highly coincident when using these two pipelines. All of the difference was investigated in discussion section.

### 2.3. The Performance in Single-Cell MDA Sequencing Data

ChimeraMiner was used to distinguish chimeras and normal reads in single-cell MDA sequencing data. We compared the chimeric rate and the amount of each type of chimera. For each single cell sample, the overall chimeric rate was between 0.93% and 4.68%, and all three types of chimeras were detected in each dataset (Table 2). 

In each single cell dataset, the inverted chimera was also the dominant type of the single-end chimera, which was coherent with the results of conventional MDA and previous works [6,8,9]. The consistent ratio between the inverted chimera and direct chimera, in all datasets, demonstrated that the application of ChimeraMiner in single cell MDA datasets was as effective as in conventional MDA datasets.

### 2.4. The Impact of Chimeras on Structural Variation

MDA1 was selected for evaluating the impact of chimeras on SV analysis. The biological sample of MDA1 is NA12878. Therefore, we deep-sequenced the unamplified genomics DNA of NA12878 to provide a control in SV analysis, named Bulk. 

We compared the number of SVs discovered before, and after, filtering chimeras to analysis the impact of chimeras on identifying SVs. The chimeras were detected using ChimeraMiner, and was filtered by using *FilterSamReads* tool of Picard [32]. After marked PCR duplicates, we used LUMPY [33] to identify SVs from the two preprocessed bam files. SVs of non-amplified data were detected as control. Then, we used SVTyper [34], to perform breakpoint genotyping of SVs. SVs, which were smaller than 50 base-pairs, and whose supported reads were smaller than 5, were filtered. From the statistical results, there is a huge difference between amplified data and non-amplified data. After filtered chimeras, the number of the false positive SVs decreased by 83.82% (Table 3). The results indicated that the recognition and filtration of chimeras abate the influence in SVs identification in MDA samples.

## 3. Discussion

In this study, we constructed ChimeraMiner for detecting chimeric read from the phi29 polymerase amplified human genome. Based on the chimeric site, located in a read or in the insertion sequence of a pair of reads, the chimeras were initially classified in single-end chimera and paired-end chimera (insertion chimera). The single-end chimera was further divided into direct chimera and inverted chimera, based on the orientation of the two subsections of the chimera. Normally, single-end chimeras were undoubtedly abandoned in almost studies. In fact, these chimeras could be converted into useful data by splitting from the chimeric sites after detection. Therefore, our study improves the utilization efficiency of the sequencing data, and is able to integrate the chimeras into the following bioinformatics analysis, rapidly. 

By comparison with previous pipelines, ChimeraMiner took less than a half the time (43.8% for MDA1 and 43.0% for MDA2) to accomplish the detection. It was mainly due to the optimization of detecting process and the different alignment tools selected. While searching for overlap sequences, ChimeraMiner used a solo cyclical alignment, but the previous pipeline utilized two nested loops. Beyond all doubt, one solo cyclical alignment took less time than the nested ones. Meanwhile, the alignment tool BWA-MEM was used in ChimeraMiner, and the previous pipeline used SOAP2. BWA-MEM was considered to take less time in paired-end reads alignment than SOAP2 does [35,36,37]. Besides, the SAM format [38] can alleviate the difficulty of the following analysis and reduce time. 

The possible generation mechanism of the two single-ended chimeras has been illustrated before in [6,8], and the ratio of these two kinds of chimeras has been calculated [6,8]. Both ChimeraMiner and the previous pipeline had similar results in the ratio of direct chimera and inverted chimera. However, no matter what kind of chimera, the absolute quantity detected by the two pipelines were different. Generally speaking, ChimeraMiner found numerous novel ones, while only a small number of chimeras were only found by using the previous pipeline. As shown in Figure 2, ChimeraMiner found 401,388 novel direct chimeras and 4,662,386 novel inverted chimeras. After detailed inspection of the previous pipeline, we found that the split-reads in the alignment results of SOAP2 were not well-handled and might be the reason of missing numerous single-end chimeras. A read which is not full-length mapped, but contains a mappable subsection, is considered to be a split-read in SOAP2. Split-reads are no doubt the candidates of single-ended chimeras for their mapping characteristic. But in alignment results of SOAP2, the unmappable parts of the split-reads were removed, where only the mapped subsections were kept. Therefore, those split-reads were ascribed as full length mapped reads and were not used for chimera detection, which caused the missing of direct and inverted chimera. After detailed investigation of MDA1, we found 6,415,806 split-reads in the alignment results by using SOAP2. By using ChimeraMiner, 258,396 of them were classified as direct chimera (64.38% of the novel direct chimeras), and 3,194,911 of them were classified as inverted chimeras (68.53% of the novel inverted chimeras). Most of the rest of the novel single-end chimeras were not discovered using the previous pipeline, due to the strict alignment condition of seed sequence, where no mismatch was allowing in seed (first 30-nt of reads) mapping. The small amount of single-ended chimeras (64,746 direct chimeras and 332,398 inverted chimeras), which were only found by using the previous pipeline, were mainly due to the different reads aligning process between BWA-MEM and SOAP2. Using ChimeraMiner, 47,934 candidate direct chimeras and 322,786 candidate inverted chimeras which could be boiled down to chimeras in the previous pipeline were filtered by the length of overlap sequences and chimera subsections. Besides, 3792 direct chimeras and 16,796 inverted chimeras which was only found in previous pipeline could map to reference with deletions, insertions and mismatches, by using BWA-MEM, and were not considered to be chimeras in ChimeraMiner. Meanwhile, randomly reports the hits of multiple aligned read in SOAP2 was another reason of quantity difference. Due to the relatively soft alignment condition of BWA, some of the reads, which were dropped using SOAP2, were mappable using BWA-MEM, and part of them (1,672,394) were ascribed as insertion chimeras. Based on the results above, ChimeraMiner showed a promoted efficiency and accuracy in handling the same data. 

MDA is highly susceptible to contamination, especially when working with single cells. Chimera formation is thought to be a problem in single-cell MDA and is potentially arisen by strand switching during the displacement process. Herein, we successfully utilized ChimeraMiner to distinguish chimeras and normal reads in the sequencing data of single-cell MDA. To the best of our knowledge, there is no systematic discovery and analysis of chimeras in single-cell MDA sequencing data previously.

After detecting chimeras in all samples, we statistically analyzed the different chimeras. First, we found that the conventional MDA (MDA1 and MDA2) generate more chimeras. It is mainly because the DNA used in the sample of MDA1 and MDA2 was lower than that in one cell, about 10% to 40% of a haploid genome. The limited amount of input DNA caused the increase of amplification duration, and the number of chimeras increased synchronously. Among single cell samples, inverted chimera was remaining the dominant type of the single-end chimera (Table 3), which is coherent with the results of conventional MDA and also the previous works [6,8,9]. The results demonstrated that applying ChimeraMiner in single cell MDA data was as affective as in conventional MDA datasets. All the results indicated that the amplified DNA, using phi29DNApol, would generate chimeras regardless of the DNA from multi-cells or single cell. 

By comparing the quantity of SVs between amplified and non-amplified genomic DNA, we known that the chimera introduced many false positive SVs. After filtering the detected chimeras, 83.82% of the false positive SVs were removed. The results indicated that the impact of chimeras on SV detection is massive, and the identification of chimeras from sequencing data, enables more accurately recognized SV. A practical SV identification pipeline is desired to be developed, based on the accurate recognition of chimeras. 

## 4. Materials and Methods 

### 4.1. Data Sources

The human genome sequence [39] was downloaded from the University of California, Santa Cruz (UCSC) Genome Browser database [40]. Assembled autosomal chromosomes, chromosome X, chromosome Y, and mitochondrial DNA were used as mapping reference (hg19). We deep-sequenced the unamplified genomics DNA of NA12878 on HiSeq platform (Illumina Inc., an Diego, CA, USA) using PE-150 mode as the control in SVs identification, and referred as Bulk.

Whole genome sequencing data, from MDA and single cell MDA, were downloaded in FASTQ format [41] from NCBI under Sequence Read Archive (SRA) accession numbers: SRX247249 and SRX252522 (referred as MDA1 and MDA2 respectively) [26]; SRR1777307 and SRR1777308 (referred as HUMDA) [42]; SRS294760 and SRS294759 (referred as BGIYH1 and BGIYH2 respectively) [43]; SRR5365373, SRR5365372, SRR5365371, SRR5365364, SRR5365363 and SRR5365362 (referred as Qiagen1, Qiagen5, Qiagen9, GE2, GE4 and GE10 respectively) [44]. The type of MDA1, MDA2 and HUMDA were PE–101, BGIYH cells were PE-100, and BJ cells were PE-150. The Table 4 provides the summary of the data; the SampleID is corresponding to the SRA accession number.

The hardware of the computing platform was four Xeon E7-4820 CPUs, 256 giga-base ram and the operating system is Ubuntu 14.04.5. In comparison, we respectively used twelve threads in the mapping step and one thread in the script of detecting chimera. The RAM used by ChimeraMiner is relatively small because of all scripts written in Perl5.18 [45].

### 4.2. Bioinformatics Analysis for Chimera Detecting

Using the pipeline of previous studies as guideline, ChimeraMiner was composed of three parts: (A) sequencing reads pre-processing, reads alignment, and filtrating; (B) unscrambling of the soft-clipped alignment reads and realignment; (C) searching overlap and achieving valid chimeras.

#### 4.2.1. Pre-Processing, Alignment, and Filtrating

**Figure 3:**
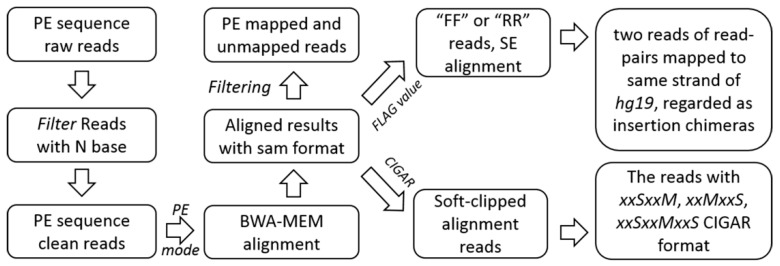
Pre-processing, alignment and filtrating. FF (forward/forward) is a pair of reads aligned in forward/forward order and RR (reverse/reverse) is a pair of reads aligned in reverse/reverse order. They were grouped into ‘not a proper pair’.

Firstly, we achieved clean reads by removing the raw reads with N base. The clean data was mapped to the human reference genome hg19 by using BWA alignment software with BWA-MEM algorithm in paired-end mode. The alignment results were saved as SAM format [38] and the aligned detail information was explored. We removed the properly paired-end aligned read pairs and unmapped reads from the SAM file. By analyzing the SAM FLAG value [38], read pairs aligned to the same strand were classified as insertion chimeras, and the soft-clipped alignment reads were retained for the following analysis by unscrambling the SAM CIGAR information [38] (Figure 3).

#### 4.2.2. Unscrambling of the Soft-Clipped Alignment Reads and Realignment

In this step, we split the soft-clipped alignment reads retained in accordance with the soft-clipped alignment locations and strand orientation, and reconstructed new paired-end files, using the parts of all soft-clipped alignment reads. 

According to the length of the chromosome, we filtered the soft-clipped sequences whose subsections were shorter than the minimum length, which was calculated by the following equation:

min (*L _soft-clipped_*) = [log_4_*L _chromosome_*] + 1 (1)

For example, the length of chromosome 1 is 249,250,621 bp; the minimum length of the soft-clipped sequences, calculated by the equation above, is 14. The soft-clipped sequences, which contained subsections shorter than a 14 bp trend to be multi-aligned on chromosome 1. Therefore, these soft-clipped sequences are removed. 

**Figure 4:**
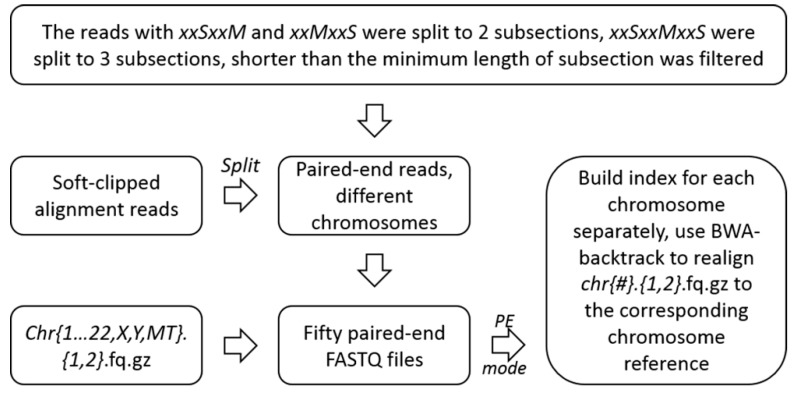
Unscrambling of the soft-clipped alignment reads and realignment.

Meanwhile, any soft-clipped sequences which contained subsections shorter than 8 bp were removed due to the large probability of multiple alignment. Then, the reconstructed data were mapped to hg19 with BWA-backtrack in paired-end mode [29]. (Figure 4). The format of reconstructed pair-end reads was show in the Figure S1 in Supplementary Materials.

#### 4.2.3. Searching Overlap and Achieving Valid Chimeras

In this step, we aimed to search the overlap sequences of split reads, and achieve the discovery of all the valid single-end chimeras. Firstly, according to the results of realignment, the recordings of the two segments mapped to different strands of the chromosome were regarded as potential inverted chimeras, and those mapped to the same strands of the chromosome, were regarded as potential direct chimeras. Then, we designed an algorithm for searching the overlap sequences of the potential chimeras. Firstly, we assumed that the overlap sequence located at the end of former subsection, and restricted the length of the overlap sequence with the maximum 30 bp. To search the overlap sequence, the last 31-nt of the former subsection were cut. If the length of the former subsection was shorter than 31-nt, we polished the subsection with N forward. Then, based on the mapped position and orientation of the following subsections, we reverse-extended the following subsection by 31-nt, from the coordinate of its first base on the reference genome. The last 31-nt of the former subsection was cyclically aligned, with the extended 31-nt of the following subsections, by shortening one nucleotide in one loop, until the overlap sequence was found or no sequence was left. We tolerated one mismatch during the cyclically alignment, as the mismatch cannot appear at the beginning of the overlap sequence (Figure S2a). Hereafter, we assumed the overlap sequence located at the head of following subsection, so we cut the first 31-nt of the following subsection and extended the former subsection by 31-nt from the coordinates of its last base on reference genome. The cyclical alignment was repeated to achieve the overlap sequence (Figure S2b). If two overlap sequences were discovered by pipeline above, the longer one was selected as the final overlap sequence. We kept the potential chimeras in which at least one overlap sequence was discovered and considered them as the valid chimeras.

## 5. Conclusions

We constructed ChimeraMiner, an improved chimeric sequence detection pipeline, based on BWA-MEM for analyzing the sequencing data of MDA, and classified the chimeric sequences into three types: Direct chimera, inverted chimera, and insertion chimera. ChimeraMiner accurately classified 24,375,539 (about 6.31%) read pairs from MDA1 and 17,480,982 (about 6.62%) read pairs from MDA2 as chimeras, spending the previous pipeline, ChimeraMiner screened 6,736,168 novel chimeras, most of which have been demonstrated to be missed by the previous pipeline. Furthermore, by applying ChimeraMiner in several single cell sequencing data, all types of chimeras were discovered in every single-cell dataset. MDA introduced plenty of false positive SVs into single cell sequencing data, and the identification and filtration of chimeras were able to remove most of the false positive SVs. ChimeraMiner revealed advanced efficiency in discovering chimeric sequences, and is promising to be widely used in single-cell sequencing to reduce the influence of chimeras to single-cell studies.

## Figures and Tables

**Table 1:** The statistics of the two chimera detection methods.

Sample	Pipelines	Times/h	Direct ^1^	Inverted ^2^	Insertion ^3^	Chimeric Rate ^4^ (%)
MDA1	previous	137	249,355	2,033,751	18,817,752	5.46
	ChimeraMiner	60	585,997	6,363,739	17,425,803	6.31
MDA2	previous	114	203,343	1,534,870	11,194,470	4.90
	ChimeraMiner	49	521,820	5,376,608	11,582,554	6.62

^1^ Direct chimera, two subsections mapped concordantly to one strand, a kind of single-end chimeras; ^2^ Inverted chimera, two subsections mapped to two reverse strands, a kind of single-end chimeras; ^3^ Insertion chimera, the chimerism happened in the sequencing insertion segments, two reads of the read-pair mapped to the same strand, also regarded as paired-end chimera; ^4^ Chimeric rate: the number of paired-end read pairs that classified as chimeras divide by the number of total paired-end read pairs.

**Table 2:** The number of the chimeras detected from single cell sequencing data.

Protocol	SampleID	Direct	Inverted	Insertion	Chimeric Rate (%)
MDA ^1^	MDA1	585,997	6,363,739	17,425,803	6.31
MDA	MDA2	521,820	5,376,608	11,582,554	6.62
scMDA ^2^	BGIYH1	240,456	1,763,085	3,334,791	2.37
scMDA	BGIYH2	64,154	598,660	1,600,853	1.42
scMDA	HUMDA	2,708,326	4,559,919	4,397,551	3.24
scMDA	Qiagen1	167,175	2,206,328	157,692	0.93
scMDA	Qiagen5	227,852	2,867,365	163,770	1.17
scMDA	Qiagen9	224,273	2,993,157	234,983	1.16
scMDA	GE2	3,336,137	10,315,253	310,998	4.38
scMDA	GE4	5,112,524	12,681,862	323,529	4.24
scMDA	GE10	4,844,590	8,899,910	387,391	4.68

^1^ MDA: conventional phi29DNApol-mediated MDA; ^2^ scMDA: single cell MDA, the genomics DNA from a single cell amplified by using phi29DNApol-mediated MDA reactions.

**Table 3:** The number of structural variations discovered from sequencing data.

SVTypes	Before Filter ^2^	After Filter ^3^	Bulk
BND ^1^	172,238	16,416	3,462
DEL	1,611	1,433	2,924
DUP	28,921	20,300	848
INV	593,858	90,726	55

^1^ BND: complex SVs, breakpoints that cannot be assigned to deletions (DEL) or duplications (DUP) or inversions (INV); ^2^ Before Filter: do not filter chimeras from mapped results; ^3^ After Filter: filter all detected chimeras from mapped results.

**Table 4:** The summary of data downloaded from NCBI.

SRA Number	Sample ID	Cell Line	Protocol	Reads’ Type
Not-released	Bulk	B-Lymphocyte ^1^	unamplified	2 × 150 bp
SRX247249	MDA1		phi29 DNA polymerase	2 × 101 bp
SRX252522	MDA2			
SRR1777307&8	HUMDA	HUVEC ^2^	phi29 DNA polymerase	2 × 101 bp
SRS294760	BGIYH1	YH ^3^	REPLI-g Mini Kit	2 × 100 bp
SRS294759	BGIYH2			
SRR5365373	Qiagen1	BJ, ATCC ^4^	REPLI-g Single Cell Kit	2 × 150 bp
SRR5365372	Qiagen5			
SRR5365371	Qiagen9			
SRR5365364	GE2	BJ, ATCC ^4^	illustra Single Cell GenomiPhiDNA Amplification Kit	2 × 150 bp
SRR5365363	GE4			
SRR5365362	GE10			

^1^ B-Lymphocyte: Peripheral vein B-Lymphocyte; ^2^ HUVEC: Human umbilical vein endothelial cell; ^3^ YH: the YH lymphoblastoid cell line; ^4^ BJ, ATCC: BJ primary human foreskin fibroblast.

## Supplementary Materials

Supplementary materials can be found at https://www.mdpi.com/1422-0067/20/8/1953/s1.

## Abbreviations

MDA	Multiple displacement amplification
WGA	Whole genome amplification
Phi29DNApol	Phi29 DNA polymerase
Chimeras	Chimeric sequence reads
SOAP2	Short Oligonucleotide Analysis Package 2
BWA	Burrows-Wheeler Aligner
SCMDA	Single cell multiple displacement amplification
Hg19	Human reference genome Feb. 2009 assembly
SRA	Sequence Read Archive
UCSC	University of California, Santa Cruz
NCBI	National Center for Biotechnology Information
B-Lymphocyte	Peripheral vein B-Lymphocyte
BJ, ATCC	BJ primary human foreskin fibroblast
HUVEC	Human umbilical vein endothelial cell
YH	YH lymphoblastoid cell line
BWA-MEM	Burrows-Wheeler Aligner-Maximal Exact Matches
SVs	Structural Variations
