# Supplementary material for: ChimeraMiner: An Improved Chimeric Read Detection Pipeline and Its Application in Single Cell Sequencing
Source: Int J Mol Sci. 2019 Apr 21;20(8):1953. doi: 10.3390/ijms20081953 (PMC6515389; doi:10.3390/ijms20081953)
Supplement: Supplementary file 1 [file ijms-20-01953-s001.pdf]

Supplementary Information

Table S1. The numerical statistics of sequenced data.

SampleID	Clean [1] Reads/M	Bases/Mbp	Mapping Ratio (%)	Depth [2] (X)	Coverage [3] (%)
Bulk	520	78,031	98.33	24.34	91.64
MDA1	773	78,031	99.10	24.63	90.95
MDA2	528	53,356	99.60	16.91	89.55
HUMDA	720	72,763	94.73	20.70	68.33
BGIYH1	451	45,072	98.93	14.05	90.02
BGIYH2	320	31,994	99.01	10.01	84.94
Qiagen1	545	81,686	94.10	22.65	79.99
Qiagen5	555	83,259	96.18	23.96	72.79
Qiagen9	598	89,636	95.49	26.30	83.55
GE2	637	95,602	91.28	24.69	82.19
GE4	854	128,053	90.67	31.70	83.48
GE10	603	90,496	95.68	23.42	69.03

[1] **Clean**: filter the reads with "N" bases. [2] **Depth**: the mean sequencing depth, the ratio of the total number of sequenced bases to the size of the reference genome. [3] **Coverage**: the proportion of sequences obtained by sequencing to the whole genome. Each sample had a high mapping ratio, revealing a well amplification during MDA.

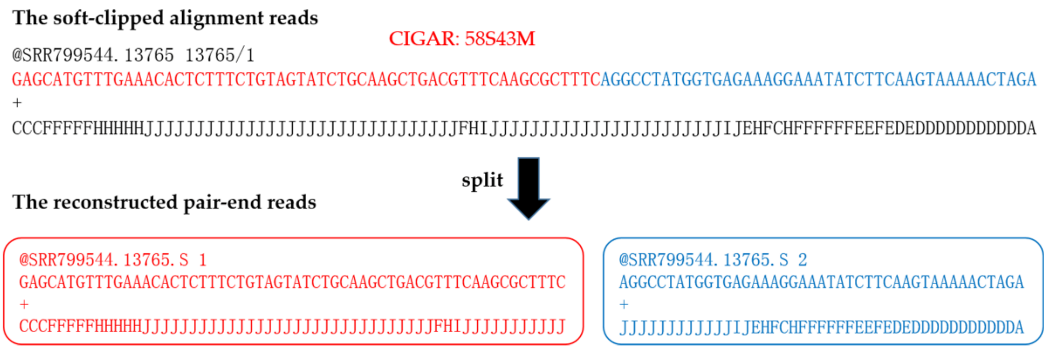

Figure S1. The format of the reconstructed pair-end file. The reads that the CIGAR is xxSxxM or xxMxxS were split to two subsections and constitute to one read-pairs. The red marked is the first subsection and referred as the read 1 of the reconstructed pair-end read, the blue marked is the second subsection and referred as the read 2 of the reconstructed pair-end read.

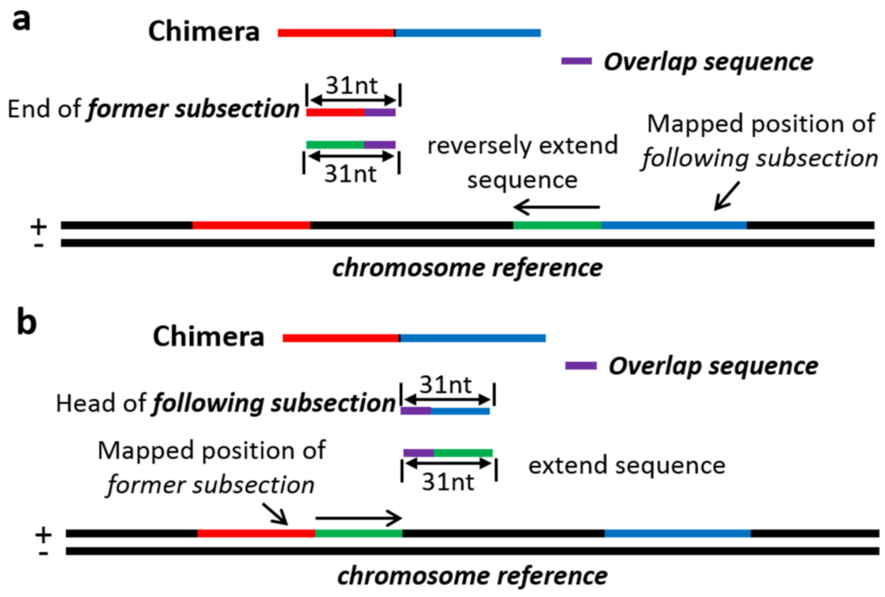

Figure S2. Demonstration of the searching overlap sequence. (a. The overlap sequence located at the end of the former subsection. b. The overlap sequence located at the head of the following subsection.) Firstly, we assumed that the overlap sequence located at the end of former subsection, and restricted the length of the overlap sequence with the maximum 30bp. To search the overlap sequence, the last 31-nt of the former subsection were cut. If the length of the former subsection was shorter than 31-nt, we polished the subsection with N forward. Then, based on the mapped position and orientation of the following subsections, we reversely extended the following subsection by 31-nt from the coordinate of its first base on reference genome. The last 31-nt of the former subsection was cyclically aligned with the extended 31-nt of the following subsections by shortening one nucleotide in one loop, till the overlap sequence was found or no sequence left. We tolerated one mismatch during the cyclically alignment, the mismatch cannot appear at the beginning of the overlap sequence (**a**). Hereafter, we assumed the overlap sequence located at the head of following subsection, so we cut the first 31-nt of the following subsection and extended the former subsection by 31-nt from the coordinates of its last base on reference genome. The cyclically alignment was repeated to achieve the overlap sequence (**b**).
